# p38α MAPK inhibition translates to cell cycle re-entry of neonatal rat ventricular cardiomyocytes and *de novo* nestin expression in response to thrombin and after apex resection

**DOI:** 10.1038/s41598-019-44712-3

**Published:** 2019-06-03

**Authors:** Vanessa Hertig, Andra Brezai, Alexandre Bergeron, Louis Villeneuve, Marc-Antoine Gillis, Angelino Calderone

**Affiliations:** 1 Montreal Heart Institute, Université de Montréal, Montréal, Québec Canada; 2 Département de Pharmacologie et Physiologie, Université de Montréal, Montréal, Québec Canada

**Keywords:** Cardiovascular biology, Cell growth

## Abstract

The present study tested the hypothesis that p38α MAPK inhibition leads to cell cycle re-entry of neonatal ventricular cardiomyocytes (NNVMs) and *de novo* nestin expression in response to thrombin and after apex resection of the neonatal rat heart. Thrombin (1 U/ml) treatment of 1-day old NNVMs did not induce cell cycle re-entry or nestin expression. Acute exposure of NNVMs to thrombin increased p38α MAPK and HSP27 phosphorylation and p38α/β MAPK inhibitor SB203580 abrogated HSP27 phosphorylation. Thrombin and SB203580 co-treatment of NNVMs led to bromodeoxyuridine incorporation and nestin expression. SB203580 (5 mg/kg) administration immediately after apex resection of 1-day old neonatal rat hearts and continued for two additional days shortened the fibrin clot length sealing the exposed left ventricular chamber. SB203580-treatment increased the density of troponin-T^(+)^-NNVMs that incorporated bromodeoxyuridine and expressed nuclear phosphohistone-3. Nestin^(+)^-NNVMs were selectively detected at the border of the fibrin clot and SB203580 potentiated the density that re-entered the cell cycle. These data suggest that the greater density of ventricular cardiomyocytes and nestin^(+)^-ventricular cardiomyocytes that re-entered the cell cycle after SB203580 treatment of the apex-resected neonatal rat heart during the acute phase of fibrin clot formation may be attributed in part to inhibition of thrombin-mediated p38α MAPK signalling.

## Introduction

The adult heart of lower vertebrates (e.g. zebrafish, newts) possess the intrinsic capacity to regenerate myocardial tissue after injury via the proliferation of pre-existing mononucleated ventricular cardiomyocytes^1–3^. In the embryonic mammalian heart, cardiomyocytes are mononucleated and re-enter the cell cycle to initiate growth^3,4^. The proliferative phenotype is retained in the neonatal mammalian heart for a brief period after birth (1–3 days) as the majority of pre-existing ventricular cardiomyocytes are mononucleated^3,5^. The latter paradigm was validated after apex-resection of the 1-day old neonatal mouse heart^5^. Apex resection of the neonatal mammalian heart initially leads to fibrin clot formation sealing the exposed left ventricular chamber followed by the deposition of extracellular matrix that persists for at least 7 days^3,5^. An ensuing inflammatory response and neovascularisation of the apex-resected ventricle provides a favourable environment for the cell cycle re-entry of pre-existing mononucleated cardiomyocytes leading to cardiac regeneration and restoration of normal ventricular function 28 days later^3,5^. Akin to the mammalian neonatal heart, a fibrin clot coincident with relatively modest cell cycle re-entry of pre-existing mononucleated cardiomyocytes was reported 7 days after amputation of the adult zebrafish heart^1,2^. At 14 days post-amputation of the zebrafish heart, robust cell cycle re-entry of mononucleated cardiomyocytes led to a significant cardiac regenerative response concomitant with fibrin clot regression^1,2^. Studies have reported that the adult rodent heart also contains a modest population of pre-existing mononucleated ventricular cardiomyocytes and exploiting their potential proliferative phenotype may represent a viable approach to initiate a partial regenerative response after ischemic injury^6,7^.

During the acute phase of healing after apex resection of the neonatal mouse heart, the protease thrombin converts fibrinogen to fibrin leading to the formation of a thrombus sealing the exposed left ventricular chamber^3,5^. In addition to fibrin clot formation; thrombin was reported to play a seminal role in scar expansion of the infarcted adult heart as pharmacological inhibition of thrombin synthesis or inactivation of the protease significantly reduced scar size^8,9^. The smaller infarct may be related in part to the attenuation of thrombin mediated recruitment of the inflammatory response post-myocardial infarction^10^. In addition, the protease directly influences cardiac remodelling as thrombin stimulation of ventricular cardiomyocytes via activation of the protease activated receptor-1 (PAR-1) induced a hypertrophic response^11,12^. Although not examined, thrombin may further prevent the cell cycle re-entry of mononucleated ventricular cardiomyocytes via recruitment of the serine/threonine kinase p38α MAPK^13,14^. Numerous studies have unequivocally revealed that pharmacological inhibition or inactivation of p38α MAPK in response to various stimuli (e.g. acidic fibroblast growth factor, interleukin-1β, neuroregulin-1 and protein kinase C) inhibits the cell cycle re-entry of neonatal and adult ventricular rodent cardiomyocytes^15–18^. Moreover, work from my lab has reported that phorbol 12,13-dibutyrate (PDBu) activation of p38α MAPK in neonatal rat ventricular cardiomyocytes prevented *de novo* expression of the intermediate filament protein nestin^15,16^. The intermediate filament protein facilitated cell cycle re-entry as shRNA-mediated depletion of constitutive nestin expression in embryonic rat ventricular cardiomyocytes or preventing induction in neonatal rat ventricular cardiomyocytes co-treated with PDBu and the p38α/β MAPK inhibitor SB203580 attenuated bromodeoxyuridine incorporation^15,16^. Based on these data, it is tempting to speculate that the local accumulation of thrombin during the acute phase of fibrin clot formation after ventricular apex resection of the neonatal heart may partially suppress cell cycle re-entry of ventricular cardiomyocytes and prevent *de novo* nestin expression via recruitment of p38α MAPK-dependent signalling events. However, directly examining the latter premise is not possible as thrombin inactivation after ventricular apex resection will prevent fibrin clot formation leading to exsanguination and death. In this regard, two complementary approaches will address the potential relationship between thrombin, p38α MAPK, cell cycle re-entry and nestin in neonatal ventricular cardiomyocytes. The first series of experiments will test the hypothesis that thrombin treatment of 1-day old neonatal rat ventricular cardiomyocytes prevents cell cycle re-entry and *de novo* nestin expression via p38α MAPK signalling. A second series of experiments will test the hypothesis that administration of the p38α/β MAPK inhibitor SB203580 during the acute phase of fibrin clot formation after ventricular apex resection of the neonatal rat heart increases the density of ventricular cardiomyocytes and subpopulation of nestin^(+)^-ventricular cardiomyocytes that re-enter the cell cycle translating to a partial cardiac regenerative response.

## Results

### Thrombin prevents cell cycle re-entry of neonatal rat ventricular cardiomyocytes and nestin expression via a p38α mapk-dependent pathway

**Figure 1:**
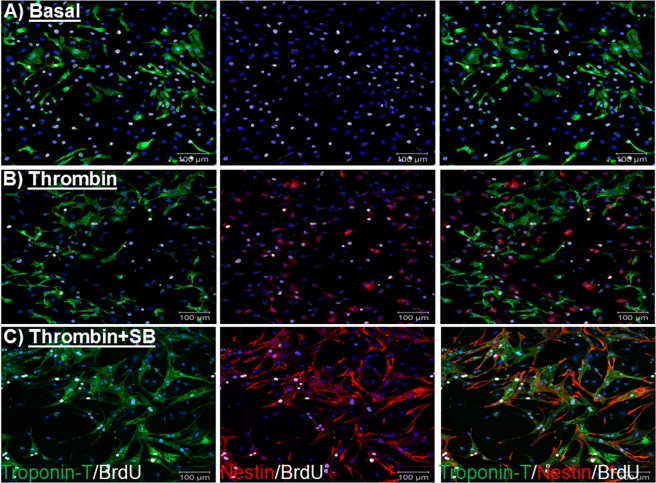
Cell cycle re-entry and *de novo* nestin expression in neonatal rat ventricular cardiomyocytes. (**A**) Modest number of untreated neonatal rat ventricular cardiomyocytes (NNVMs) delineated by cardiac troponin-T staining (green fluorescence) re-entered the cell cycle as determined by bromodeoxyuridine (BrdU; grey fluorescence) incorporation. Furthermore, nestin staining (red fluorescence) of NNVMs was not observed. (**B**) Three day treatment with thrombin (1 U/ml) failed to increase cell cycle re-entry in NNVMs as depicted by BrdU incorporation and nestin staining was absent. By contrast, nestin staining detected in cells lacking troponin-T immunoreactivity were identified as neonatal rat ventricular fibroblasts (See Supplemental Fig. 2). (**C**) The co-treatment with the p38α/β MAPK inhibitor SB203580 (SB; 10 µM) and thrombin for three days promoted cycle re-entry and *de novo* nestin expression in NNVMs. DAPI staining identifies the nucleus (blue fluorescence).

**Figure 2:**
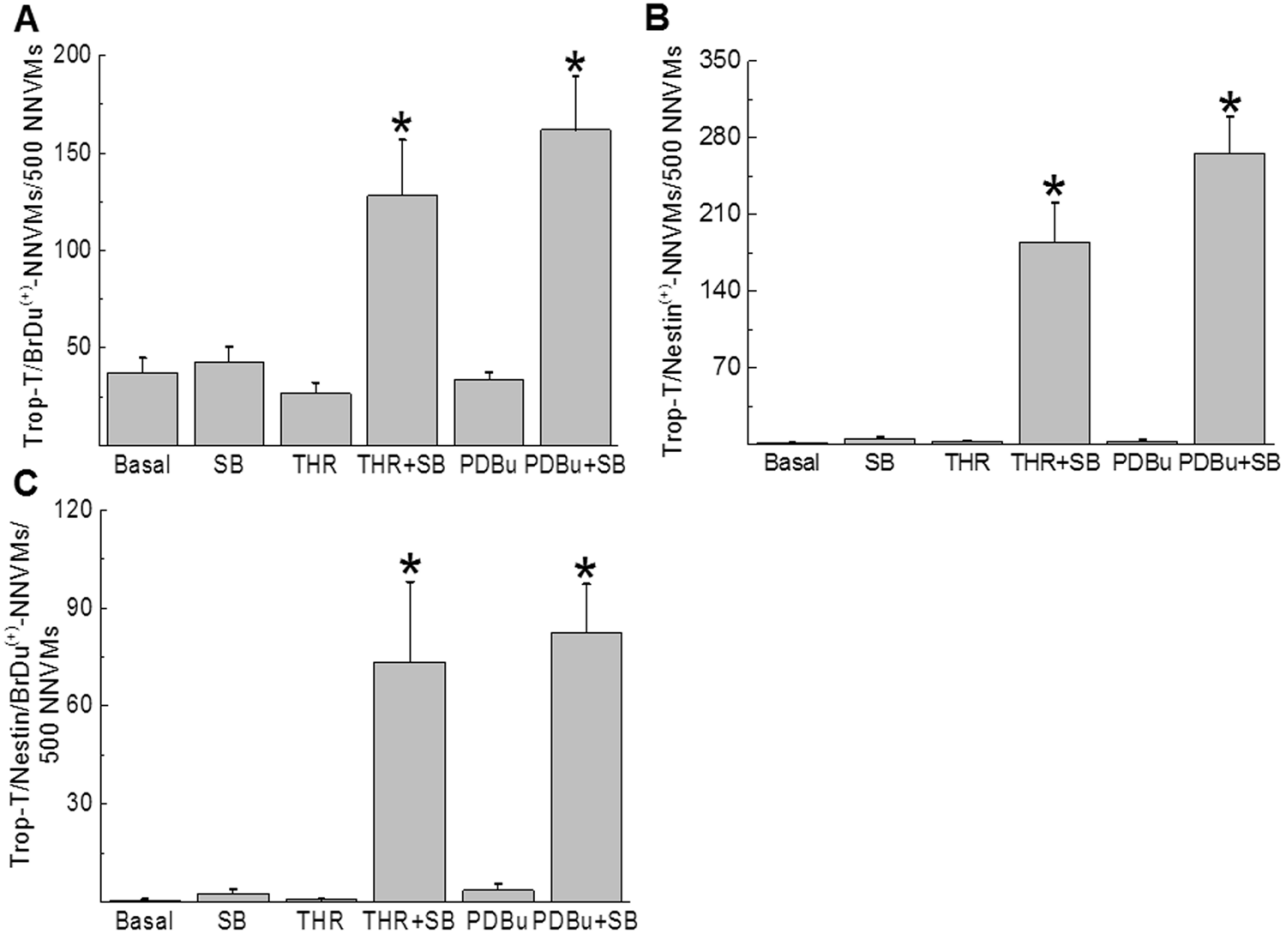
Quantitative assessment of cell cycle re-entry and *de novo* nestin expression in NNVMs. (**A**) Graph represents a quantitative analysis of the density of cardiac troponin-T^(+)^-NNVMs that re-entered the cell cycle characterized by BrdU incorporation. (**B**) Graph represents a quantitative analysis of the density of cardiac troponin-T^(+)^-NNVMs that co-expressed nestin. (**C**) Graph represents a quantitative analysis of the density of cardiac troponin-T^(+)^-NNVMs that co-expressed nestin and incorporated BrdU. Experiments were performed on *n* = 5 independent preparations of neonatal ventricular cells and (*) denotes *P* < 0.05 versus thrombin or PDBu alone.

**Figure 3:**
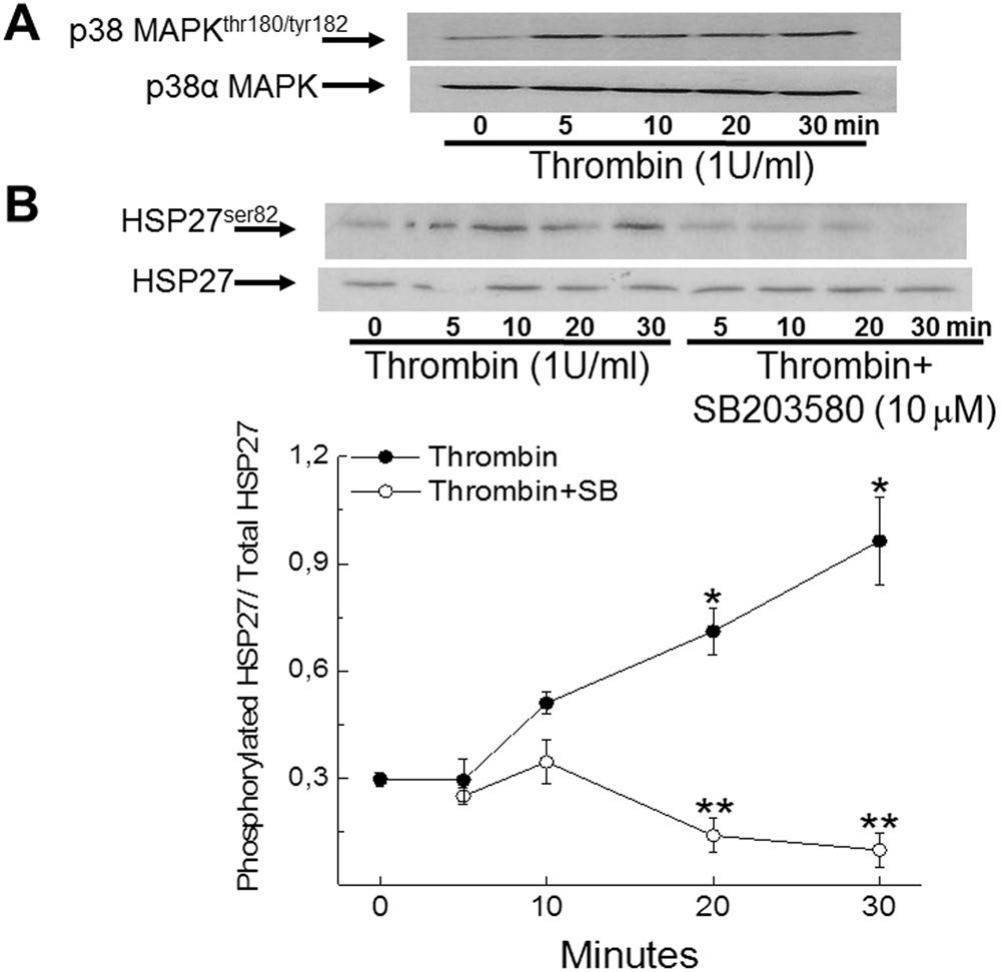
Thrombin stimulation of neonatal rat ventricular cells recruits p38α MAPK signaling. (**A**) The acute treatment of neonatal ventricular cells with thrombin (1 U/ml) increased phosphorylation of the threonine^180^/tyrosine^182^ residues of p38 MAPK (n = 2 independent experiments). (**B**) The acute treatment of neonatal ventricular cells with thrombin (1 U/ml) significantly increased phosphorylation of the serine^82^ residue of heat shock protein (HSP27; 27 kDa) (n = 3 independent experiments) at 20 and 30 minutes. Co-treatment with the p38α/β MAPK inhibitor SB203580 (SB; 10 µM) abrogated thrombin-mediated phosphorylation of HSP27 (n = 3 independent experiments). (*) Denotes *P* < 0.05 versus basal, (**) denotes *P* < 0.05 versus thrombin and p38 MAPK and HSP27 phosphorylation data was normalized to total p38α MAPK and HSP27 protein content.

As previously reported, ventricular cells isolated from 1-day old neonatal rat hearts consist predominantly of mononucleated ventricular cardiomyocytes and a modest population of ventricular fibroblasts (∼15–20%)^15,16^. In the absence of stimulation for a period of three days, cardiac troponin-T staining of neonatal rat ventricular cardiomyocytes (NNVMs) revealed that a modest number re-entered the cell cycle, as depicted by bromodeoxyuridine incorporation (Figs 1A and 2A). The treatment with thrombin (1 U/ml) once every 24 hours for three consecutive days did not increase the number of cardiac troponin-T^(+)^-NNVMs that incorporated bromodeoxyuridine (Figs 1B and 2A). In parallel, treatment with the protein kinase C activator phorbol 12,13-dibutyrate (PDBu; 100 nM) for three days likewise failed to increase the number of cardiac troponin-T^(+)^-NNVMs that re-entered the cell cycle (Supplemental Figs 1A and 2A). The acute exposure (0–30 minutes) of neonatal ventricular cells with thrombin (1 U/ml) significantly increased p38α MAPK phosphorylation (n = 2) and phosphorylation of the putative downstream target heat shock protein 27 (HSP27;n = 3) (Fig. 3A,B)^19^. In the presence of the p38α/β MAPK inhibitor SB203580 (10 µM), thrombin phosphorylation of HSP27 at 20 and 30 minutes was completely suppressed (Fig. 3B). Pre-treatment with SB203580 (10 µM) prior to thrombin exposure for a period of three days robustly increased the number of cardiac troponin-T^(+)^-ventricular cardiomyocytes that incorporated bromodeoxyuridine as compared to NNVMs treated alone with thrombin (Figs 1C and 2A). Significant cell cycle re-entry was likewise observed in cardiac troponin-T^(+)^-ventricular cardiomyocytes following the co-treatment with SB203580 and PDBu (100 nM) for a period of three days (Supplemental Figs 1B and 2A).

**Figure 4:**
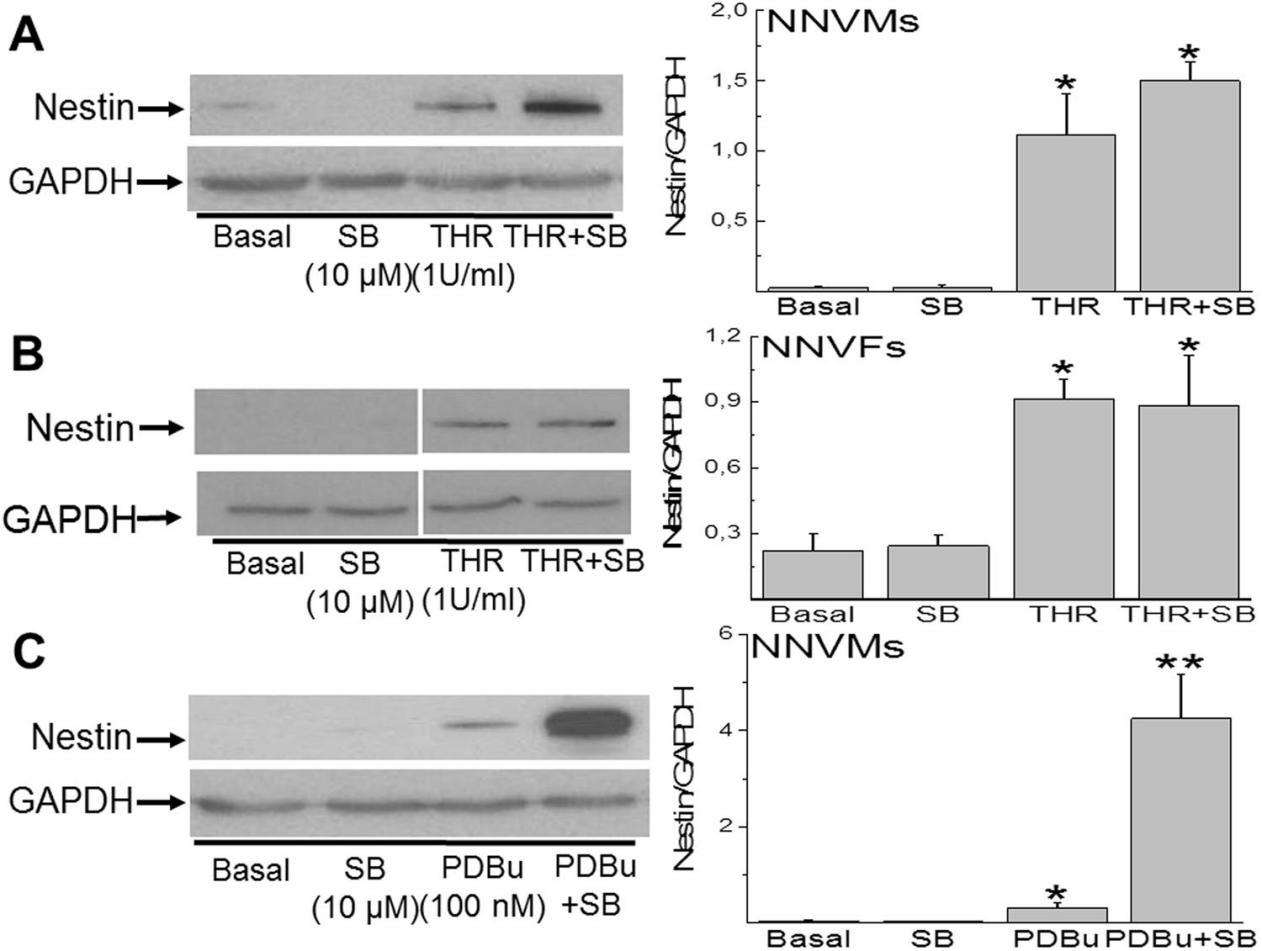
Nestin expression in neonatal rat ventricular cells. (**A**) Thrombin (THR; 1 U/ml) treatment of neonatal rat ventricular cells for three days increased nestin protein levels and co-treatment with SB203580 (SB; 10 µM) non-significantly increased the response. The increased protein levels of nestin after thrombin and SB203580 co-treatment was attributed to the *de novo* expression of the intermediate filament protein in neonatal rat ventricular cardiomyocytes (See Fig. 1C). (**B**) Thrombin (1 U/ml) treatment of 1^st^ passage neonatal rat ventricular fibroblasts for 24 hours increased nestin protein levels and co-treatment with SB203580 (SB; 10 µM) did not suppress expression. (**C**) Phorbol 12,13-dibutyrate (PDBu; 100 nM) treatment of neonatal rat ventricular cells for three days increased nestin protein levels and co-treatment with SB203580 (SB; 10 µM) potentiated the response. The increased protein levels of nestin after PDBu and SB203580 co-treatment was attributed to the *de novo* expression of the intermediate filament protein in neonatal rat ventricular cardiomyocytes (See Supplemental Fig. 1). In each Western blot performed, nestin (~220 kDa) and GAPDH (~37 kDa) data represent images cropped from the same gel. Experiments were performed on *n* = 4 independent preparations of neonatal ventricular cells and *n* = 5–6 independent preparations of neonatal ventricular fibroblasts, (*) denotes *P* < 0.05 versus basal, (**) denotes *P* < 0.05 comparing PDBu/SB203580 versus PDBu alone and the nestin signal was normalized to GAPDH.

Previous work from my lab reported that the cell cycle re-entry of NNVMs after p38α MAPK inhibition occurred in part via *de novo* expression of the intermediate filament protein nestin^15,16^. In untreated cardiac troponin-T^(+)^-ventricular cardiomyocytes, nestin immunoreactivity was absent (Figs 1A and 2B) whereas the intermediate filament protein was detected in collagen type I^(+)^-neonatal rat ventricular fibroblasts (Supplemental Fig. 2). Following a three day treatment of neonatal ventricular cells with thrombin (n = 4), nestin protein levels were upregulated (Fig. 4A) and immunofluorescence revealed that the intermediate filament protein was detected preferentially in neonatal rat ventricular fibroblasts whereas the intermediate filament protein was not expressed in cardiac troponin-T^(+)^-NNVMs (Figs 1B and 2B). Consistent with the former observation, a 24 hour treatment of 1^st^ passage neonatal ventricular fibroblasts with thrombin (1 U/ml) significantly increased nestin protein levels (n = 5–6) (Fig. 4B). The pre-treatment of neonatal ventricular cells with SB203580 (10 µM) prior to thrombin administration for a period of three days further increased nestin protein levels (Fig. 4A) (n = 4) and immunofluorescence revealed *de novo* expression of the intermediate filament protein in cardiac troponin-T^(+)^-ventricular cardiomyocytes (Figs 1C and 2B). By contrast, the co-treatment of neonatal rat ventricular fibroblasts with SB203580 and thrombin did not potentiate nestin protein levels (n = 5–6) (Fig. 4B). Analogous to thrombin, PDBu (100 nM) treatment of neonatal ventricular cells for three days induced nestin expression exclusively in ventricular fibroblasts (n = 4), as previously reported^20^. The co-treatment of ventricular cells with SB203580 and PDBu treatment for three days significantly upregulated nestin protein levels (Fig. 4C) attributed primarily to the *de novo* expression of the intermediate filament protein in cardiac troponin-T^(+)^-ventricular cardiomyocytes (n = 4) (Supplemental Figure 2B & Figure 2B). Lastly, in the presence of SB203580, ∼72% of cardiac troponin-T^(+)^-ventricular cardiomyocytes that incorporated bromodeoxyuridine in response to thrombin (Figs 1C and 2C) or PDBu (Supplemental Fig. 2B,C) co-expressed the intermediate filament protein nestin.

### Morphological and cellular remodelling of the apex-resected neonatal rat heart; impact of the p38α/β mapk inhibitor sb203580

**Figure 5:**
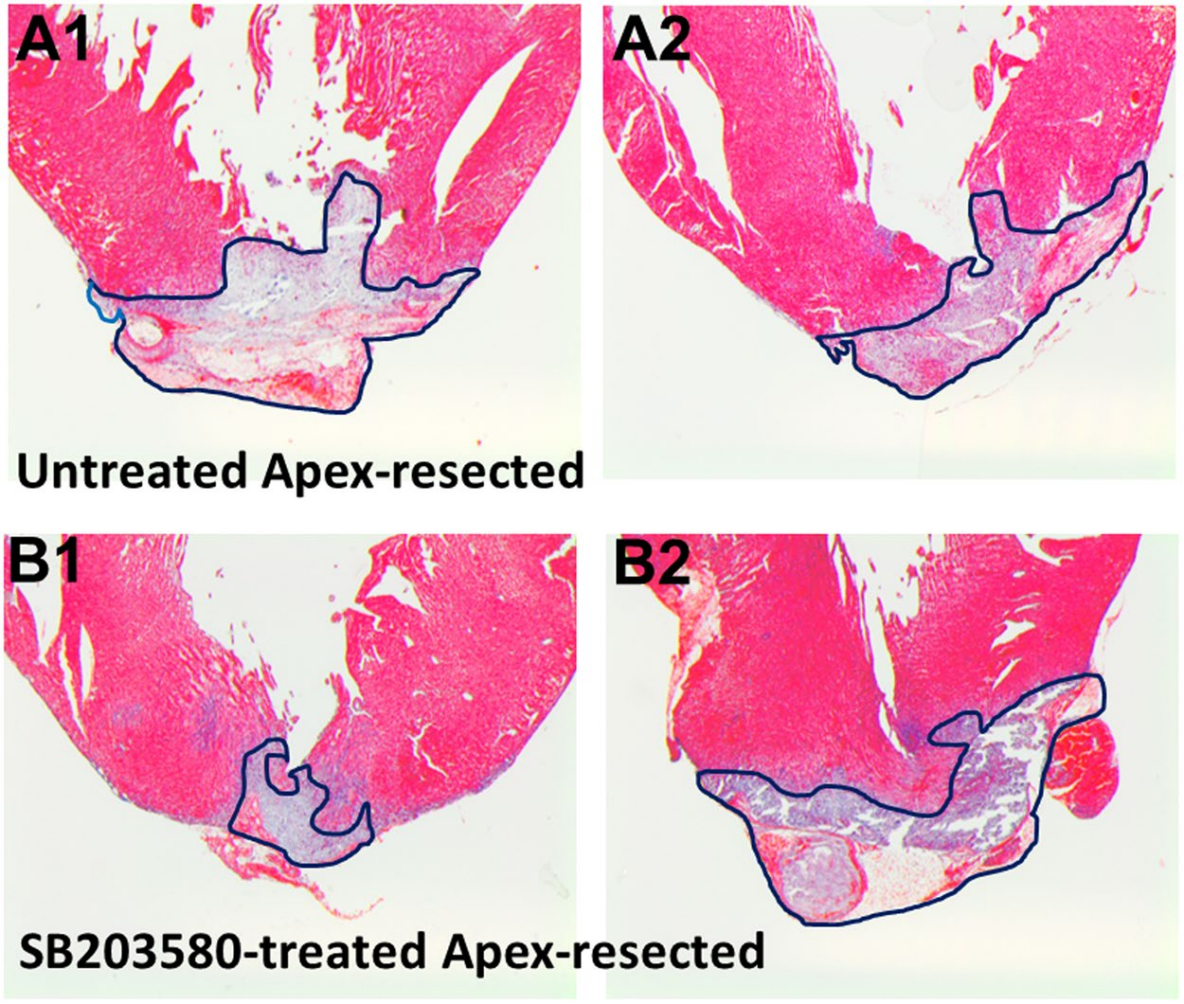
Immunohistochemical analysis of apex-resected 1-day old neonatal rat pups. (**A1**,**A2**) Three days after apex resection of vehicle-treated neonatal rat pups (n = 5), Masson trichrome staining revealed the presence of a large fibrin clot sealing the exposed left ventricular chamber. (**B1**,**B2**) Following administration of the p38α/β MAPK inhibitor SB203580 (5 mg/kg) to apex-resected neonatal rat hearts (n = 6), the overall fibrin clot region was smaller but did not reach statistical significance. However, the fibrin clot length sealing the exposed left ventricular chamber appeared to be reduced. In the SB203580-treated neonatal rat pup depicted in panel B2, the exposed left ventricular chamber was completely sealed with myocardial tissue and a large fibrin clot persisted. The blue line demarcates the fibrin clot region.

**Figure 6:**
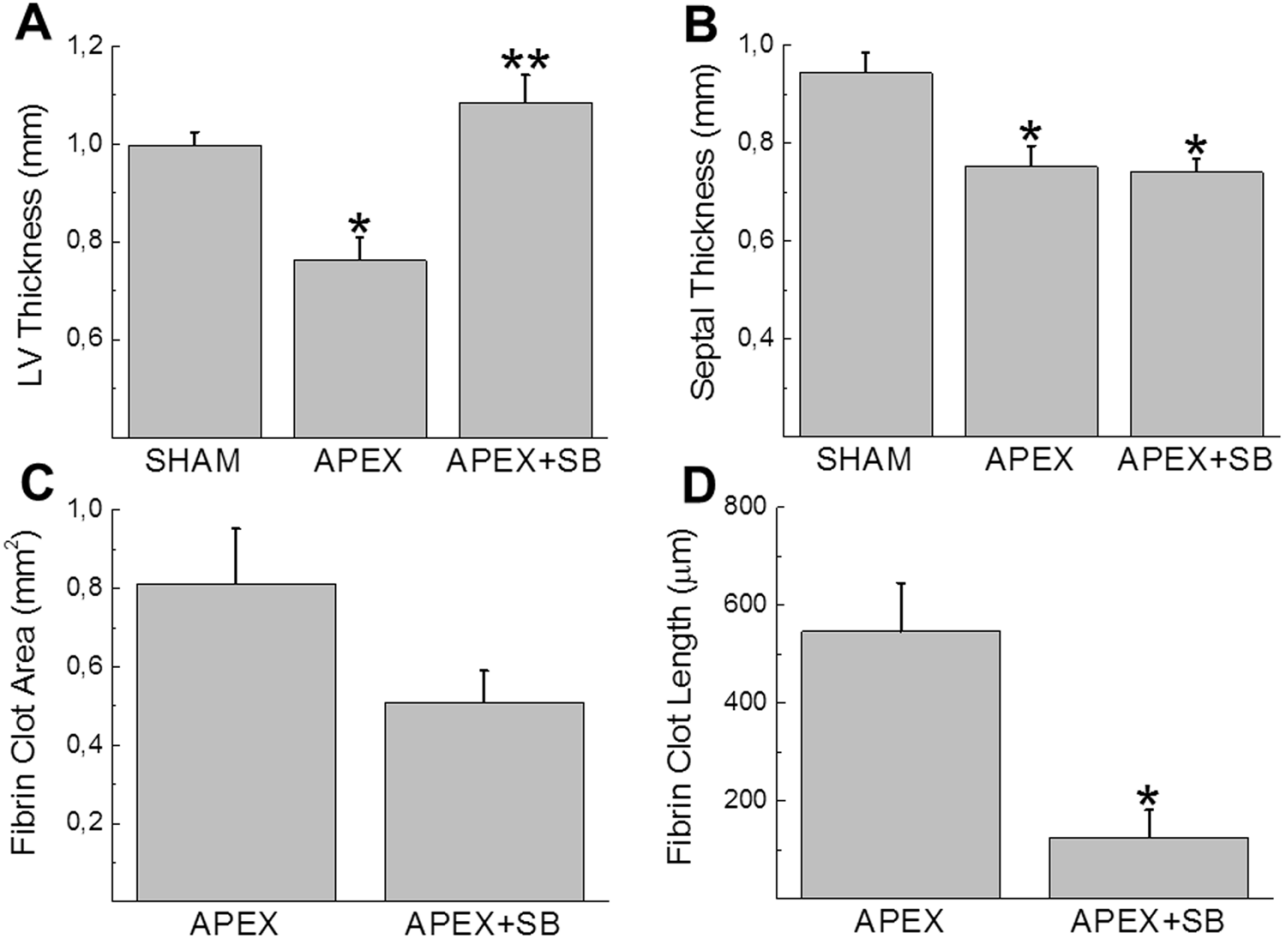
Morphology of the apex-resected neonatal rat heart. (**A**,**B**) As compared to sham rat pups (n = 5), left ventricular and septal wall thickness were significantly reduced after apex resection (APEX; n = 5). SB203580 (SB; 5 mg/kg) treatment of apex-resected neonatal rat pups (APEX + SB; n = 6) selectively restored left ventricular wall thickness. (**C**) Masson trichrome staining revealed that the fibrin clot area was reduced in SB203580-treated apex-resected neonatal rat pups, but did not reach statistical significance as compared to vehicle-treated apex-resected pups. (**D**) Immunofluorescence approach delineating ventricular cardiomyocytes by cardiac troponin-T staining revealed that SB203580 treatment of apex-resected hearts (n = 6) significantly shortened the fibrin clot length sealing the exposed left ventricular chamber as compared to vehicle-treated apex-resected hearts (n = 5). (*) Denotes *P* < 0.05 versus sham rats or vehicle-apex-resected hearts and (**) denotes *P* < 0.05 versus vehicle-treated apex-resected hearts.

The observation that thrombin prevents cell cycle re-entry of NNVMs provided the rationale to inactivate the protease during the acute phase of fibrin clot formation after apex resection of the neonatal rat heart to elucidate a potential role in the cell cycle re-entry of NNVMs. However, thrombin inactivation after successful ventricular apex resection will prevent fibrin clot formation leading to exsanguination and death. Nonetheless, the seminal role of p38α MAPK preventing the cell cycle re-entry of NNVMs in response to thrombin provided the impetus to pharmacologically target the serine/threonine kinase after apex resection of the neonatal rat heart. Immediately following successful ventricular apex resection of 1-day old neonatal rat hearts, minute bleeding was observed. In the absence of bleeding, neonatal rat pups that underwent surgery were eliminated from the study. Three days after apex resection, a fibrin clot that completely sealed the exposed left ventricular chamber was evident in each neonatal rat pup that underwent surgery (Fig. 5A1,A2). In vehicle-treated apex-resected neonatal rat hearts (n = 5), left ventricular free wall and septal thickness were significantly reduced as compared to sham neonatal rats (n = 6) (Fig. 6A,B). p38α/β MAPK inhibitor SB203580 (5 mg/kg) administered immediately after apex resection and continued for two additional days reduced the overall fibrin clot region (0,509 ± 0,083 mm^2^; n = 6) but did not reach statistical significance (p = 0,08) versus vehicle-treated apex-resected neonatal rats (0,812 ± 0,141 mm^2^; n = 5) (Figs 5 and 6C). However, the fibrin clot length spanning the exposed left ventricular chamber appeared to have shortened in SB203580-treated apex-resected neonatal rat hearts (Fig. 5B1,B2). The immunohistochemical approach did not provide the resolution required to precisely measure the fibrin clot length. Thus, an immunofluorescence approach with a greater resolution was used to determine the fibrin clot length and NNVMs were identified by cardiac troponin-T staining. Consistent with the immunohistochemical data, the fibrin clot area was smaller in SB203580-treated apex resected hearts (0,339 ± 0,101 mm^2^; n = 6) albeit the reduction was not statistically significant (p = 0,10) compared to vehicle-treated apex-resected hearts (0,745 ± 0,216 mm^2^; n = 5). In vehicle-treated apex-resected neonatal hearts, the range of the fibrin clot length sealing the exposed left ventricular chamber was 219 to 769 µm with an average length of 546 ± 154 µm (n = 5) (Fig. 6D). In SB203580-treated apex-resected neonatal rat hearts, the range of fibrin clot length sealing the exposed left ventricular chamber was 0 to 333 µm and the average length of 125 ± 46 µm (n = 6) was significantly (p < 0,05) shorter compared to vehicle-treated apex-resected neonatal rat hearts (Fig. 6D). In a SB203580-treated apex-resected neonatal rat heart in which the left ventricular chamber was completely sealed with myocardial tissue, a large prominent fibrin clot persisted (Fig. 5B2). Lastly, the shortened fibrin clot length sealing the exposed chamber of SB203580-treated apex-resected neonatal rat hearts led to a recovery of left ventricular free wall thickness (Fig. 6A).

**Figure 7:**
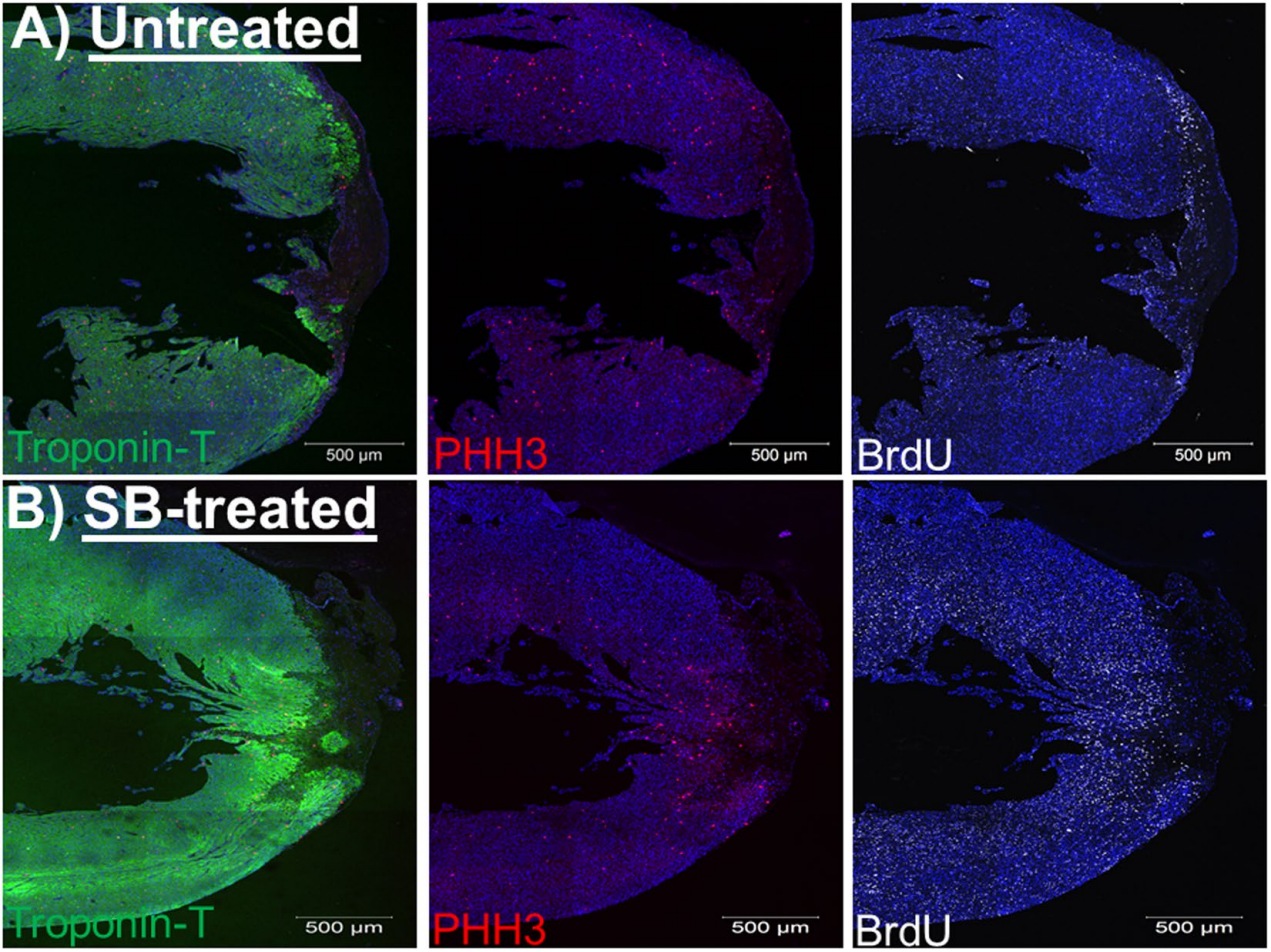
Bromodeoxyurdine incorporation and phosphohistone-3 staining in the viable myocardium of the apex-resected region of untreated- and SB203580-treated rat hearts. (**A**) In vehicle-treated apex-resected neonatal hearts (n = 4), cardiac troponin-T staining (green fluorescence) of ventricular cardiomyocytes revealed the presence of a significantly large fibrin clot sealing the exposed left ventricular chamber. Phosphohistone-3 (PHH3; red fluorescence) and bromodeoxyuridine (BrdU; grey fluorescence) incorporation was detected adjacent to the fibrin clot and in the viable myocardium of the resected apex. (**B**) In the SB203580-treated apex-resected neonatal heart, cardiac troponin-T staining of ventricular cardiomyocytes revealed that the exposed left ventricular chamber was almost completely sealed. Phosphohistone-3 (PHH3) and bromodeoxyuridine (BrdU) incorporation was detected adjacent to the fibrin clot and in the viable myocardium of the resected apex. DAPI staining identifies the nucleus (blue fluorescence).

**Figure 8:**
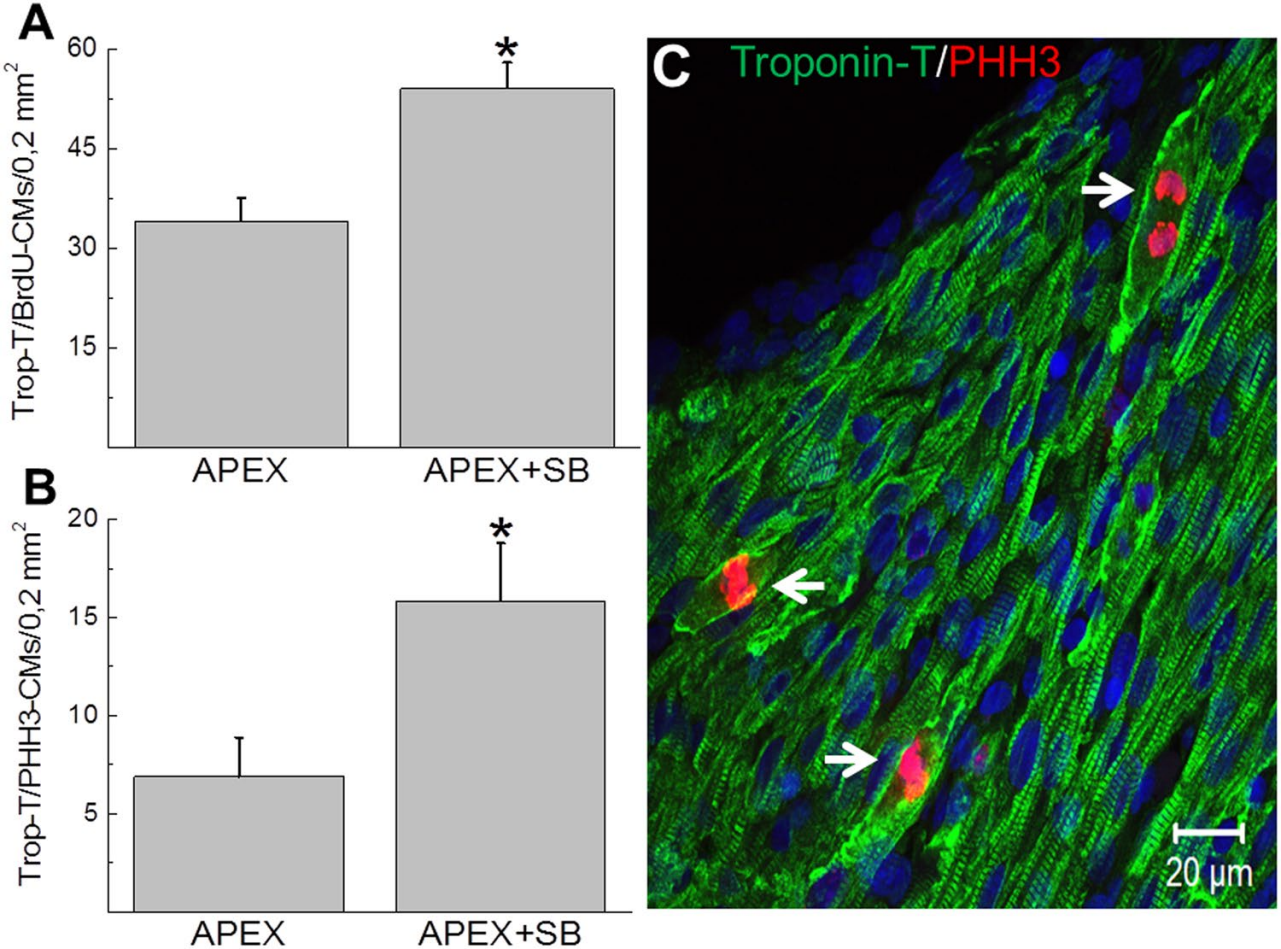
Quantitative assessment of cell cycle re-entry of NNVMs identified in the viable myocardium of the apex-resected region. The density of cardiac troponin-T (Trop-T) ventricular cardiomyocytes that incorporated (**A**) bromodeoxyuridine (Brdu) and (**B**) expressed phosphohistone-3 (PHH3) were significantly increased in SB203580-treated apex-resected neonatal hearts (APEX + SB; n = 5–6) compared to vehicle-treated apex-resected hearts (APEX; n = 4–5). (*) Denotes *P* < 0.05 versus untreated apex-resected hearts. (*) Denotes *P* < 0.05 versus vehicle-treated apex-resected hearts. (**C**) In SB203580-treated apex-resected neonatal hearts, marginalization of cardiac troponin-T to the periphery was detected in numerous ventricular cardiomyocytes concomitant with nuclear expression of the G2-M marker phosphohistone-3 (indicated by arrow). An analogous phenotype was identified in the vehicle-treated apex-resected neonatal rat heart. DAPI staining identifies the nucleus (blue fluorescence).

The shorter fibrin clot length of SB203580-treated apex-resected neonatal hearts is suggestive of a partial cardiac regenerative response initiated by mononucleated ventricular cardiomyocytes. Cell cycle re-entry of neonatal cardiomyocytes into the S-phase was assessed via incorporation of bromodeoxyuridine and progression to G2-M phase confirmed with the nuclear marker phosphohistone-3. In vehicle-treated apex-resected neonatal hearts (n = 4), cardiac troponin-T/bromodeoxyuridine^(+)^-ventricular cardiomyocytes were identified in the viable myocardium (maximum distance of ∼500 µm from the fibrin clot) of the apex region (Figs 7A and 8A). In SB203580-treated apex-resected neonatal hearts (n = 5), the density of cardiac troponin-T/bromodeoxyuridine^(+)^-ventricular cardiomyocytes in the viable myocardium of the apex region was significantly (p < 0,05) increased as compared to untreated apex-resected neonatal hearts (Figs 7B and 8A). Cell cycle progression of cardiac troponin-T^(+)^-ventricular cardiomyocytes into G2-M phase delineated by nuclear staining of phosphohistone-3 was identified in the viable myocardium of the apex region of untreated apex-resected hearts (n = 5) (Figs 7A and 8B). SB203580 treatment of apex-resected hearts (n = 6) significantly (p < 0,05) upregulated the density of cardiac troponin-T^(+)^-ventricular cardiomyocytes that co-expressed nuclear phosphohistone-3 (Figs 7B and 8B). Furthermore, a morphological phenotype consistent with cell cycle re-entry was identified in vehicle-treated and SB203580-treated apex-resected neonatal hearts as a subpopulation of ventricular cardiomyocytes expressing nuclear phosphohistone-3 was characterized by marginalization of cardiac troponin-T to the periphery (Fig. 8C)^5^.

**Figure 9:**
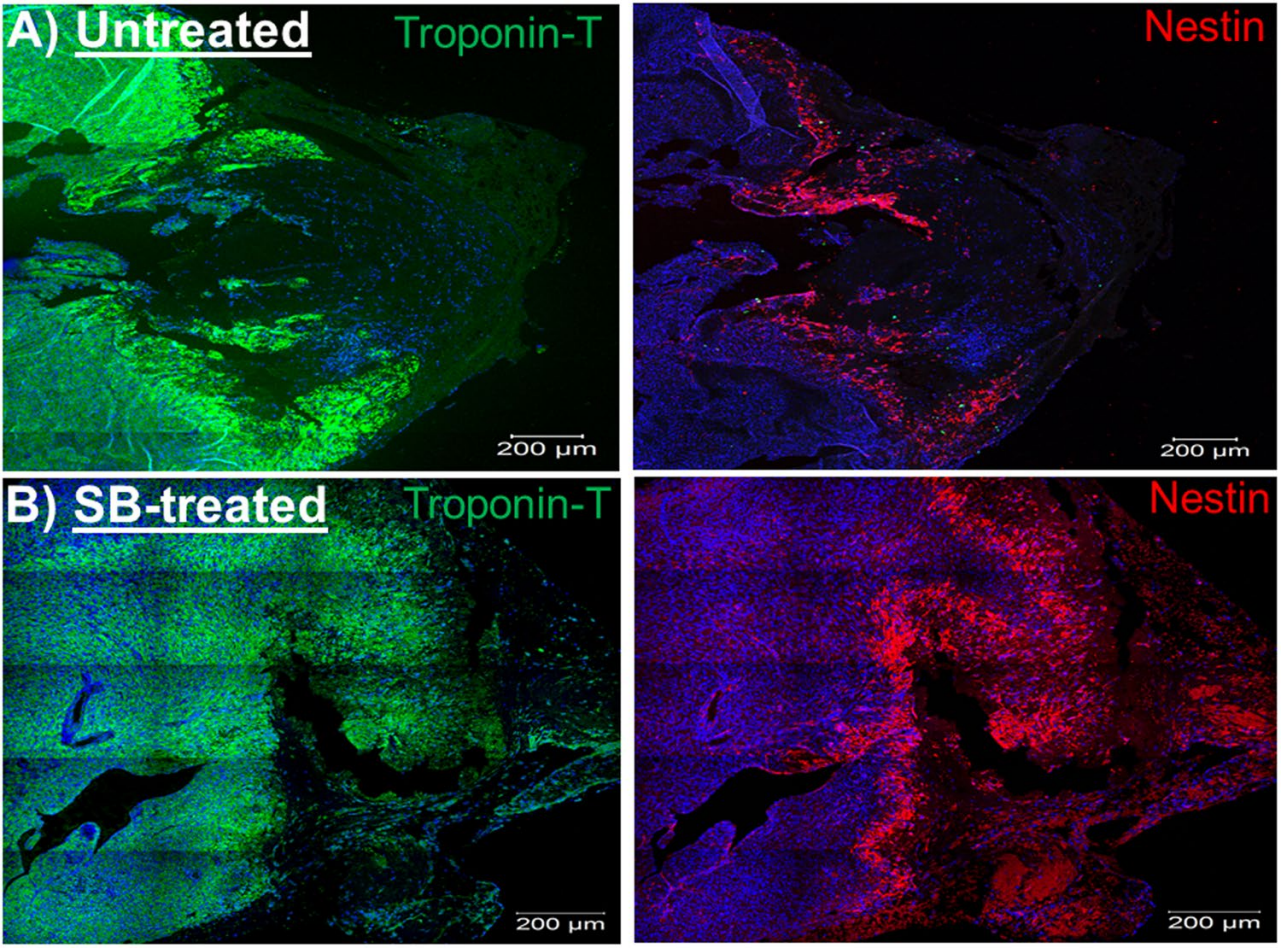
The predominant appearance of nestin^(+)^-cells along the border of the fibrin clot. (**A**) In a vehicle-treated apex-resected heart, a large fibrin clot sealing the exposed left ventricular chamber was evident. Furthermore, a population of nestin^(+)^-cells (red fluorescence) was detected bordering the entire length of the fibrin clot region. (**B**) In a SB203580-treated apex-resected heart, the resected left ventricle was completely sealed with myocardial tissue characterized by the appearance of cardiac troponin-T^(+)^-NNVMs. Furthermore, an important population of nestin^(+)^-cells (red fluorescence) was still prevalent juxtaposed to the fibrin clot. DAPI staining identifies the nucleus (blue fluorescence).

**Figure 10:**
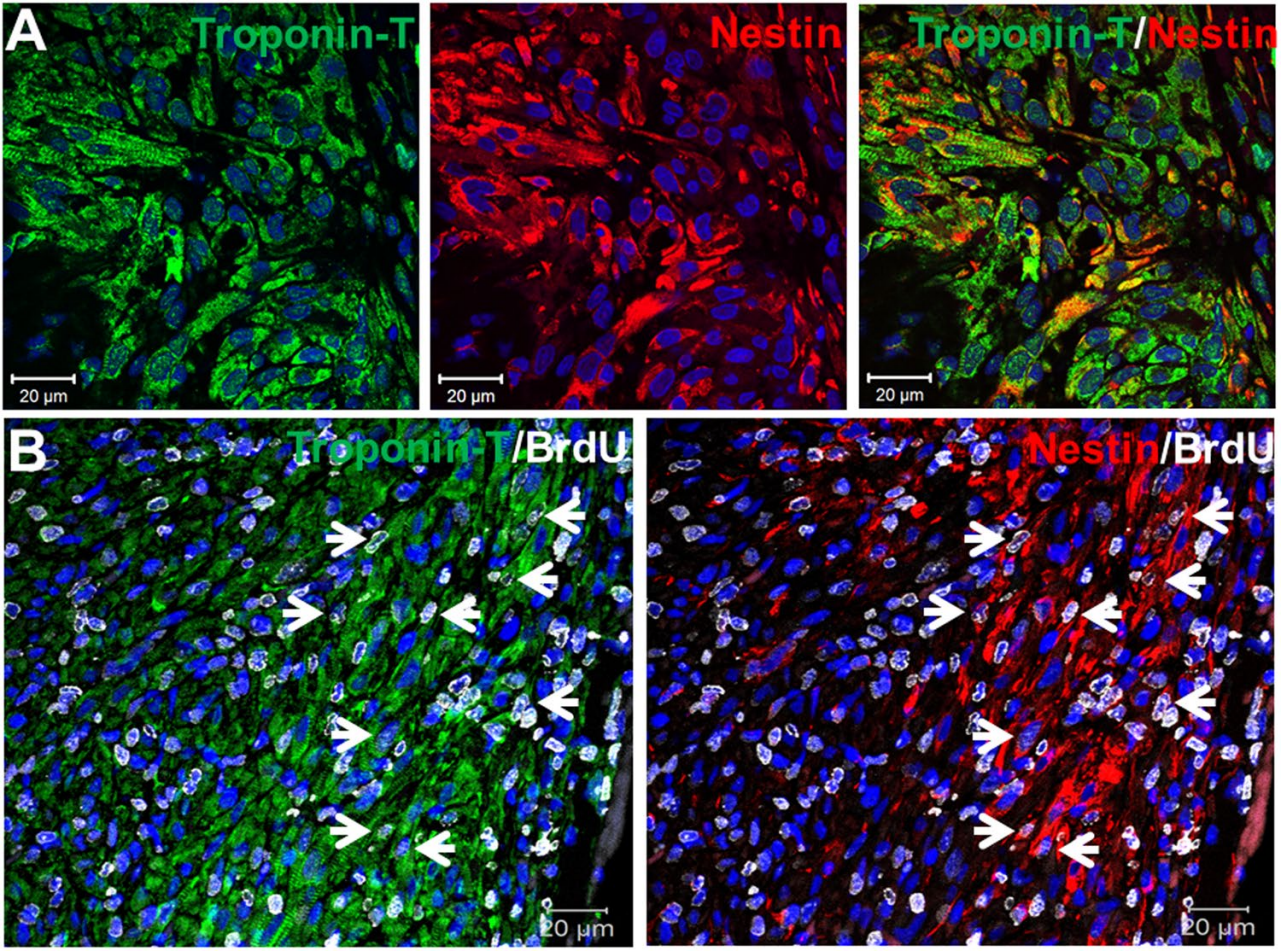
Nestin/cardiac troponin-T^(+)^-ventricular cardiomyocytes were detected exclusively adjacent to the fibrin clot region and a subpopulation re-entered the cell cycle. (**A**) The majority of nestin^(+)^-cells (red fluorescence) identified adjacent to the fibrin clot region were neonatal ventricular cardiomyocytes characterized by the co-expression of cardiac troponin-T (green fluorescence). (**B**) In SB203580-treated apex-resected hearts, a subpopulation of cardiac troponin-T/nestin^(+)^-neonatal ventricular cardiomyocytes bordering the fibrin clot region re-entered the cell cycle (indicated by arrow) characterized by bromodeoxyuridine (Brdu; grey fluorescence) incorporation. Cell cycle re-entry was also observed in cardiac troponin-T/nestin^(+)^-neonatal ventricular cardiomyocytes along the border of the vehicle-treated apex-resected heart (see Fig. 11). DAPI staining identified the nucleus (blue fluorescence).

**Figure 11:**
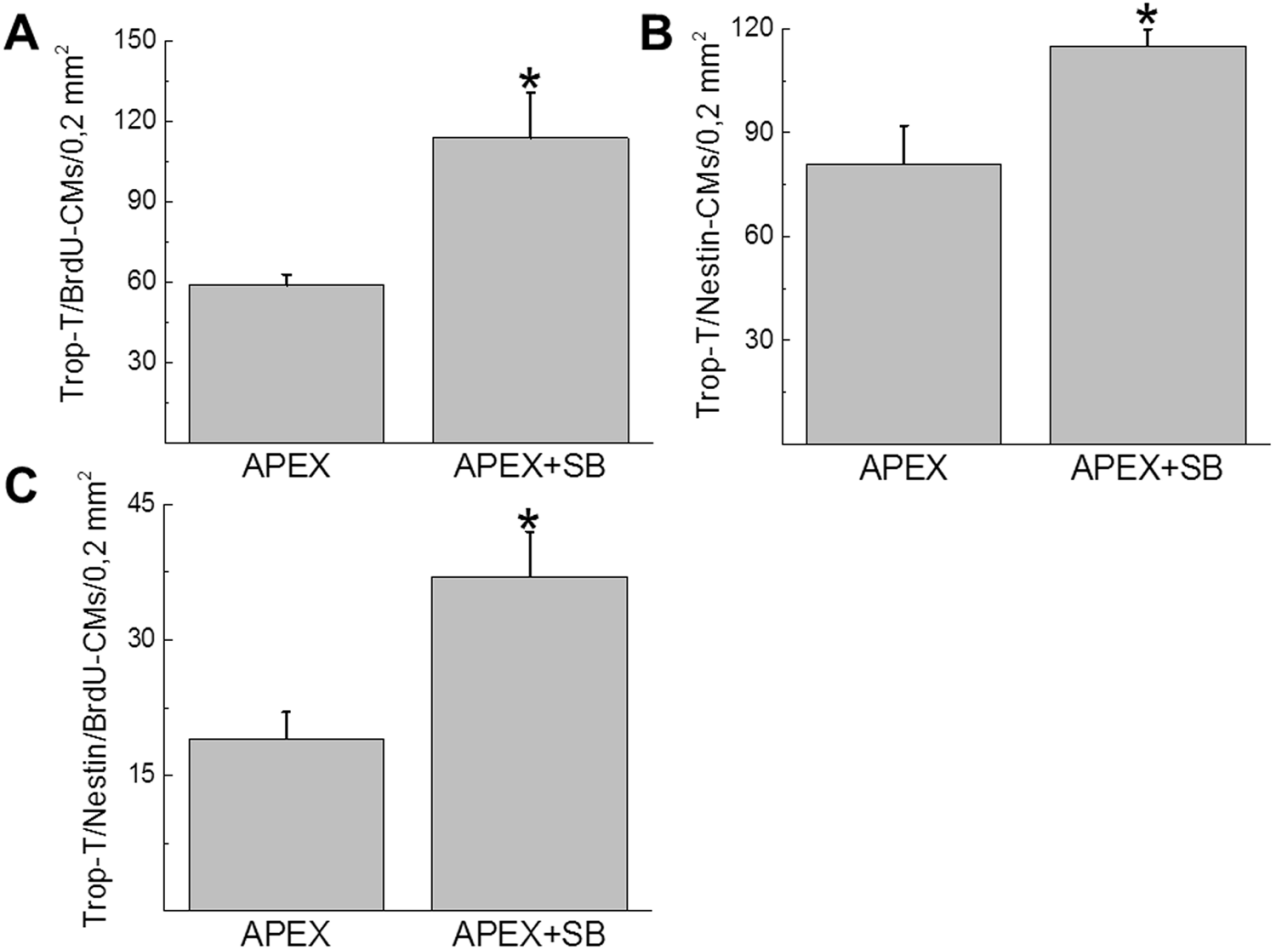
Quantitative assessment of cell cycle re-entry of NNVMs and nestin^(+)^-NNVMs residing selectively adjacent to the fibrin clot region of the apex-resected heart. (**A**) The density of cardiac troponin-T^(+)^-ventricular cardiomyocytes bordering the fibrin clot region that incorporated bromodeoxyuridine was significantly greater in SB203580-treated apex-resected neonatal hearts (APEX + SB; n = 5) compared to vehicle-treated apex-resected hearts (APEX; n = 4). (**B**,**C**) The density of cardiac troponin-T^(+)^-ventricular cardiomyocytes adjacent to the fibrin clot that co-expressed nestin and concomitantly incorporated bromodeoxyuridine (BrdU) were significantly increased in SB203580-treated apex-resected neonatal hearts (APEX + SB; n = 5) compared to vehicle-treated apex-resected hearts (APEX; n = 4). (*) Denotes *P* < 0.05 versus vehicle-treated apex-resected hearts.

Previous work from my lab and others have reported that the reparative fibrotic response of the infarcted adult rodent heart was associated with the appearance of a modest number of pre-existing cardiomyocytes that expressed the intermediate filament protein nestin residing preferentially at the peri-infarct region^15,21^. In vehicle-treated and SB203580-treated apex-resected neonatal hearts, a significant population of nestin^(+)^-cells was detected along the border of the entire length of the fibrin clot (Fig. 9A,B). The predominant population of nestin^(+)^-cells detected at the border of the fibrin clot were cardiac troponin-T^(+)^-cardiomyocytes co-expressing the intermediate filament protein (Fig. 10A,B). However, nestin staining was not exclusive to cardiomyocytes at the border of the fibrin clot region as the intermediate filament protein was also detected in non-cardiomyocytes, albeit identify was not determined. Based on previous studies published by my lab, non-cardiomyocytes that express nestin during cardiac remodelling of the infarcted rodent heart include resident neural crest-derived neural progenitor/stem cells and/or activated ventricular fibroblasts^20,21^. With regard to cell cycle re-entry, the population of cardiac troponin-T^(+)^-ventricular cardiomyocytes detected at the border of the fibrin clot that incorporated bromodeoxyuridine was significantly greater in SB203580-treated apex-resected hearts (n = 5) compared to vehicle-treated apex-resected neonatal hearts (n = 4) (Fig. 11A). In vehicle-treated apex-resected neonatal hearts (n = 4), a subpopulation of cardiac troponin-T^(+)^-ventricular cardiomyocytes that co-expressed nestin re-entered the cell cycle characterized by bromodeoxyuridine incorporation (Fig. 11B). In SB203580-treated apex-resected hearts (n = 5), the density of cardiac troponin-T^(+)^-ventricular cardiomyocytes that co-expressed nestin and concomitantly incorporated bromodeoxyuridine at the border of the fibrin clot was significantly (p < 0,05) greater compared to untreated apex-resected hearts (Figs 10B and 11C). Collectively, these data suggest that the shortened fibrin clot length of SB203580-treated apex-resected neonatal hearts may be attributed in part to a cardiac regenerative response secondary to the greater density of ventricular cardiomyocytes and subpopulation of nestin^(+)^-ventricular cardiomyocytes that re-entered the cell cycle.

## Discussion

In the infarcted adult rabbit heart, increased expression of the transmembrane receptor tissue factor in ventricular cardiomyocytes bordering the ischemically damaged region initiated VII/VIIa mediated accumulation of the protease thrombin at the site of injury^9^. The latter study established a biological role as pharmacological inhibition of tissue factor or hirudin inhibition of activated thrombin reduced infarct size^9^. A significant reduction of infarct size was also reported in apolipoprotein E-deficient/LDL receptor-deficient adult male mice treated with the activated thrombin inhibitor melagatran after exposure of the heart to acute hypoxic stress^22^. Consistent with the latter paradigm, pharmacological inhibition of thrombin activation of the protease-activated receptor-1 (PAR-1) limited scar expansion of the adult rodent heart after ischemia/reperfusion injury^23,24^. In contrast to the established pharmacological data, PAR-1 deficiency failed to reduce infarct size and administration of PAR-1 agonists did not further increase the scar region after ischemic damage^24,25^. It has been suggested that the disparate pharmacological and transgenic effect on infarct size may be attributed in part to off-target actions of PAR-1 antagonists^24^. Alternatively, protease-activated receptor-4 (PAR-4) was reported to compensate for PAR-1 deficiency as pharmacological inhibition or transgenic depletion of PAR-4 reduced infarct size in the ischemically damaged adult heart^24^. Based on these observations, the local accumulation of thrombin during the acute phase of fibrin clot formation after apex resection of the left ventricle of the neonatal rat heart may directly influence cardiomyocyte responsiveness after injury. Indeed, the reported recruitment of p38α MAPK signalling after thrombin stimulation of neonatal ventricular cardiomyocytes may prevent cell cycle re-entry after apex resection^11,12^. Engel and colleagues demonstrated that p38α MAPK activity was significantly repressed during the embryonic proliferative phase of heart development and expression of a dominant-negative form of the serine/threonine kinase increased cell cycle re-entry and cytokinesis of fetal cardiomyocytes delineated by nuclear phosphohistone-3 staining^17^. In neonatal rodent ventricular cardiomyocytes, pharmacological suppression of p38α MAPK with the inhibitor SB203580 potentiated cell cycle re-entry and cytokinesis in response to diverse stimuli including acidic fibroblast growth factor, IL-1β, neuroregulin-1 and phorbol ester recruitment of protein kinase C-dependent pathways^15–17^. The latter paradigm was preserved in the infarcted adult mouse heart as treatment with SB203580 alone was sufficient to increase the density of ventricular cardiomyocytes that re-entered the cell cycle^18^. An analogous mechanism is prevalent during regeneration of the injured adult zebrafish heart as p38α MAPK inactivation was identified as a seminal prerequisite event initiating the migration of mononucleated cardiomyocytes to the damaged region and subsequent cell cycle re-entry^1,2^. In the present study, the absence of an increase in cell cycle re-entry of cardiac troponin-T^(+)^-neonatal ventricular cardiomyocytes after thrombin treatment was associated with the acute recruitment of p38α MAPK signalling delineated by the increased phosphorylation of the serine/threonine kinase and an enhanced phosphorylated state of the putative downstream target HSP27. Pre-treatment with the p38α/β MAPK inhibitor SB203580 abrogated thrombin stimulated HSP27 phosphorylation and concomitantly increased the number of cardiac troponin-T^(+)^-neonatal ventricular cardiomyocytes that incorporated bromodeoxyuridine. Cell cycle re-entry of fetal chicken cardiomyocytes cultured in three-dimensional myocardial tissue was also reported after thrombin activation of PAR-1^26^. However, the latter study did not examine the downstream signalling events coupling PAR-1 activation to cell cycle re-entry^26^. Thus, the *in vitro* data highlight a novel antiproliferative role of thrombin and further suggests that the local accumulation of the protease in the apex-resected neonatal heart or infarcted adult heart may in part suppress the cell cycle re-entry of mononucleated ventricular cardiomyocytes^6,7,15^.

Previous studies from my lab and others have reported the appearance of the intermediate filament protein nestin in pre-existing ventricular cardiomyocytes selectively bordering the infarct region of the ischemically damaged human and rodent heart^27,28^. The latter response recapitulated in part an embryonic phenotype as nestin was expressed in proliferating cardiomyocytes during embryogenesis and depletion of the intermediate filament protein in rat embryonic ventricular cardiomyocytes suppressed cell cycle re-entry^15,16,29^. Despite expression of the intermediate filament protein, only a modest number of nestin^(+)^-ventricular cardiomyocytes were detected in the infarcted adult heart and cell cycle re-entry was not observed^15^. The latter paradigm may be attributed in part to the recruitment of p38α MAPK in the infarcted adult heart as work from my lab revealed that pharmacological inhibition of the serine/threonine kinase concomitant with phorbol 12,13-dibutyrate stimulation of neonatal rat ventricular cardiomyocytes led to *de novo* nestin expression and cell cycle re-entry^15,16^. Moreover, an AAV9-shRNA approach revealed that preventing *de novo* nestin expression in neonatal rat ventricular cardiomyocytes co-treated with PDBu and the p38α/β MAPK inhibitor SB203580 attenuated cell cycle re-entry^16^. Based on these observations, the relationship between thrombin, p38α MAPK, cell cycle re-entry and nestin was explored in neonatal rat ventricular cardiomyocytes. The treatment of neonatal rat ventricular cells with thrombin for 3 days increased nestin protein levels albeit expression of the intermediate filament protein was induced exclusively in neonatal ventricular fibroblasts. The latter response may have occurred via thrombin activation of PAR-1 expressed in ventricular fibroblasts^30^. In vascular smooth muscle cells and radial glial cells, thrombin stimulation likewise increased expression of the intermediate filament protein nestin^31,32^. Moreover, thrombin mediated nestin upregulation in ventricular fibroblasts may contribute in part to the acquisition of an activated phenotype identified in the infarcted adult heart during reparative fibrosis^20^. By contrast, p38α MAPK inhibition prior to thrombin administration was required to initiate *de novo* nestin expression in neonatal ventricular cardiomyocytes. In addition, ∼72% of neonatal ventricular cardiomyocytes that expressed nestin in response to SB203580/thrombin co-treatment incorporated bromodeoxyuridine further supporting our previous *in vitro* observation that the intermediate filament protein drives in part cell cycle re-entry^15,16^. Thus, the *in vitro* data reported in NNVMs indirectly suggests that the reported local accumulation of the protease thrombin at the border of the infarct region in the ischemically damaged adult heart may have contributed in part to the modest appearance of nestin-expressing ventricular cardiomyocytes and concomitant failure to re-enter the cell cycle.

The study by Porello and colleagues reported that following ventricular apex resection of the 1-day old neonatal mouse heart, a complete regenerative was observed 28 days post-surgery^5^. By contrast, Andersen and colleagues revealed that apex resection was associated with a modest number of cardiomyocytes that re-entered the cell cycle translating to significant scarring in the absence of a regenerative cardiac response^33^. The study by Bryant *et al*. suggested that apex resection of a large region of the neonatal mouse heart may in part compromise the cardiac regenerative response^34^. Despite these discordant findings, a salient feature of healing after apex resection of the neonatal heart or the adult zebrafish heart is the formation of a thrombus that seals the exposed left ventricular chamber^1,3,5,34^. In the present study, successful ventricular apex resection of 1-day old neonatal rat hearts was confirmed by the appearance of minute bleeding immediately after surgery leading to the formation of a fibrin clot sealing the exposed chamber. The latter paradigm may further represent an essential prerequisite biological event initiating a cardiac regenerative response as fibrin is an important natural scaffold providing the necessary matrix for cell migration, adhesion and proliferation^35^. Morphologically, fibrin clot formation after apex resection was associated with a significant reduction of left ventricular free wall thickness and septal wall thickness in vehicle-treated apex-resected heart as compared to neonatal sham hearts. Within the remaining viable myocardium of the apex region and at the border of the fibrin clot, a subpopulation of cardiac troponin-T^(+)^-ventricular cardiomyocytes re-entered S-phase and progressed to the G2-M phase characterized by nuclear staining of exogenously administered bromodeoxyuridine and phosphistone-3, respectively. Furthermore, a subpopulation of ventricular cardiomyocytes identified in vehicle-treated apex-resected hearts that expressed nuclear phosphohistone-3 was characterized by the marginalization of cardiac troponin-T to the periphery^5^. The acute phase of healing after apex resection was further highlighted by the novel appearance of a population of cycling ventricular cardiomyocytes along the entire length of the fibrin clot that expressed the intermediate filament protein nestin. The latter response was analogous to that observed in the infarcted adult rodent heart^21,27^. However, in contrast to the infarcted adult heart, the response was robust and nestin^(+)^-ventricular cardiomyocytes bordering the fibrin clot of the vehicle-treated apex resected neonatal heart re-entered the cell cycle^15^. Collectively, these data are consistent with the preponderance of previously published studies demonstrating that fibrin clot formation secondary to apex resection of the neonatal rodent heart was associated with the cell cycle re-entry of ventricular cardiomyocytes^3,5,34,36^.

The observation that thrombin acting via p38α MAPK prevented the cell cycle re-entry of neonatal rat ventricular cardiomyocytes and *de novo* nestin expression provides the impetus to inactivate the protease during the acute phase of fibrin clot formation after apex resection of the neonatal rat heart to assess a potential role in cardiac regeneration. However, protease inactivation after successful ventricular apex resection will prevent fibrin clot formation leading to exsanguination and death. Nonetheless, the seminal role of p38α MAPK preventing the appearance of cycling nestin^(+)^- neonatal rat ventricular cardiomyocytes in response to thrombin provided the rational to pharmacologically target the serine/threonine kinase after apex resection. Previous studies have reported that pharmacological inhibition of p38α MAPK signaling in the infarcted adult heart improved left ventricular function and limited adverse cardiac remodeling after ischemic injury^37–39^. In the present study, SB203580 treatment of apex-resected hearts reduced the fibrin clot area but did not reach statistical significance. However, the fibrin clot length sealing the exposed left ventricular chamber was significantly shortened and left ventricular free wall thickness was restored in SB203580-treated apex-resected hearts compared to vehicle-treated apex-resected hearts. Concomitant with shortening of the fibrin clot length, SB203580 treatment of apex-resected hearts potentiated the density of cycling ventricular cardiomyocytes in the viable myocardium of the apex region and at the border of the fibrin clot. Furthermore, consistent with the *in vitro* data, SB203580-mediated inhibition of p38α MAPK signalling increased the density of neonatal rat ventricular cardiomyocytes bordering the fibrin clot region that expressed nestin and concomitantly re-entered the cell cycle. Collectively, these findings suggest that the significantly greater increase of cycling ventricular cardiomyocytes and nestin^(+)^-ventricular cardiomyocytes after p38α MAPK inhibition may have accelerated the regenerative response during the acute phase of healing after apex resection characterized by the significant shortening of the fibrin clot length. Thus, the local accumulation of thrombin and the potential recruitment of additional stimuli acting via p38α MAPK may collectively delay the cardiac regenerative response of the neonatal rat heart during the acute phase of fibrin clot formation. Based on these data, recruitment of p38α MAPK-dependent signalling by diverse stimuli including G-protein coupled receptors in the infarcted adult mammalian heart may play a seminal role limiting the appearance and suppressing the cell cycle re-entry of nestin^(+)^-ventricular cardiomyocytes bordering the scar^15,16,21,28,38–40^. 

## Methods

### Ethics Approval

The use and care of laboratory rodents was according to the Canadian Council for Animal Care and approved by the Animal Care Committee of the Montreal Heart Institute.

### Apex resection of 1-day old neonatal rat pups

1-Day old neonatal Sprague-Dawley rat pups (Charles River, Canada) were anesthetized for 5–6 minutes on ice. A thoracotomy was performed and the heart remained within the thorax during apex resection of the left ventricle. Successful apex resection of each rat pup was confirmed by the presence of minute bleeding. Thereafter, the thorax was sutured with 5–0 silk (Johnson & Johnson, Markham, Ontario) and rat pups were placed on a heating pad and exposed to a heating lamp to initiate recovery. In the absence of minute bleeding, neonatal rats that underwent surgery were eliminated. Following apex resection, neonatal rat pups were arbitrarily selected and injected IP with either the vehicle or the p38α/β MAPK inhibitor SB203580 (5 mg/kg) immediately after surgery and administered once daily for two additional days. SB203580 was dissolved in 500 µL of the vehicle containing DMSO (5 µL);polyethyleneglycol (150 µL);tween (25 µL); saline (320 µL). Each rat pup received 50 µL of the vehicle or SB203580 delivered with a BD U-100 insulin needle. In parallel, 200 µL of bromodeoxyuridine (200 mg/kg; Sigma-Aldrich) was injected IP immediately after surgery and administered once daily for two additional days. Bromodeoxyuridine was dissolved in saline, pH adjusted to 7,4 and filtered prior to injection. Three days after surgery, rat pups were sacrificed, the heart excised, washed in saline and fixed in formalin for immunohistochemistry and immunofluorescence analysis.

### Neonatal rat ventricular cells; cardiomyocytes and fibroblasts

Neonatal rat ventricular cells were isolated from 1-day old Sprague-Dawley rat pups (sacrificed by decapitation) (Charles River, Canada) as previously described^15,16^. Ventricular cells were plated at a density of 400 cells/mm^2^ in DMEM-low glucose (Hyclone Laboratories, Logan, UT) supplemented with 7% heat-inactivated FBS and 1% penicillin-streptomycin for 48 hours, subsequently washed and maintained in DMEM-low glucose containing insulin (5 μg/ml), transferrin (5 μg/ml), and selenium (5 ng/ml) (ITS; BD Bioscience, Bedford, MA) for 24–36 hours prior to the experimental protocol. As previously reported, 80–85% of ventricular cells are cardiomyocytes characterized by cardiac troponin-T staining whereas ventricular fibroblasts constitute 15–20% of the population delineated by collagen type I staining (Supplemental Fig. 2)^15^. In immunofluorescence experiments, ventricular cells were plated on poly-D-lysine coated glass coverslips in 12-well plates. In Western blot experiments, neonatal rat ventricular cells were plated in p100 plates.

Neonatal rat ventricular cells plated in p12-well plates or p100 plates were treated for a period of 72 hrs with thrombin (1 U/ml; Sigma-Aldrich, St. Louis, MO), p38α/β MAPK inhibitor SB203580 (10 μM; LC Laboratories, Woburn, MA), thrombin and SB203580, phorbol 12, 13-dibutyrate (PDBu, 10^−7^ M; Sigma-Aldrich,), or PDBu and SB203580. The p38α/β MAPK inhibitor SB203580 was added 15 minutes prior to thrombin or PDBu and re-administered 24 and 48 hours after the initial treatment. To assess cell cycle re-entry, bromodeoxyuridine (BrdU; 20 μM; Sigma-Aldrich) was added to neonatal rat ventricular cells seeded in 12-well plates during the last 24 hrs of the experimental protocol. The number of cardiac troponin-T^(+)^- ventricular cardiomyocytes that incorporated BrdU and co-expressed nestin was counted and normalized to a total of 500 ventricular cardiomyocytes. Neonatal rat ventricular fibroblasts characterized by the absence of cardiac troponin-T staining were collagen type I-immunoreactive and expressed nestin (Supplemental Fig. 2)

Neonatal rat ventricular fibroblasts were isolated and cultured as previously described^20^. 1^st^ passaged fibroblasts (150–200 cells/mm^2^) were plated in DMEM-low glucose (Hyclone laboratories, Logan, UT) supplemented with 7% heat-inactivated FBS and 1% penicillin-streptomycin for 36–48 hours, subsequently washed and maintained for 36–48 hours in DMEM-low glucose media supplemented with insulin (5 μg/ml), transferrin (5 μg/ml), and selenium (5 ng/ml) (BD bioscience, Bedford, MA) prior to the experimental protocol. Thereafter, neonatal rat ventricular fibroblasts were exposed to thrombin (1 U/ml; Sigma-Aldrich) alone or co-treated with SB203580 (10 μM) for a period of 24 hrs.

### Immunofluorescence & immunohistochemistry

Neonatal rat ventricular cells were plated on glass coverslips coated with poly-D-lysine and at the end of the experimental protocol fixed with 2% paraformaldehyde^15,16^. Prior to immunofluorescence, ventricular cells were treated with 2M HCl for 15 minutes to denature DNA to detect BrdU incorporation. Primary antibodies used include mouse monoclonal anti-nestin (1:400; Chemicon, Temicula, CA), rabbit polyclonal anti-cardiac troponin-T (1:200; Abcam, Cambridge, UK), chicken polyclonal anti-Brdu (1:500; Abcam) and rabbit polyclonal anti-collagen type I (1:400; Abcam). The data was normalized to a total of 500 neonatal ventricular cardiomyocytes delineated by cardiac troponin-T staining. Neonatal rat ventricular fibroblasts were identified with a rabbit polyclonal anti-collagen type I (1:200; Abcam).

The neonatal mouse heart was removed, fixed in formalin and longitudinal tissue sections (5–10 μm thickness) were prepared^20^. Tissue sections were subsequently dehydrated and embedded in paraffin. With regard to the analysis of the fibrin clot area and left ventricular thickness; at least 3–4 serial sections of each paraffin-fixed neonatal rat heart were stained with Masson’s trichrome and analyzed^20^. Images were captured with the Olympus QICAM colour video camera interfaced with an Olympus CKX41 microscope and the Olympus Stream Basic Image Analysis Software (Center Valley, PA) was used to calculate the fibrin clot region and morphology of the viable myocardium (left ventricular wall thickness and septal wall thickness). Prior to immunofluorescence, tissue sections were deparaffinised, hydrated and exposed to the antigen retrieval method (10 mM sodium citrate, pH 6) at a temperature of 100 °C for 30 minutes^15,16,20^. Primary antibodies used include mouse monoclonal anti-nestin (1:150; Chemicon), mouse monoclonal anti-cardiac troponin-T (1:300; Abcam), rabbit polyclonal anti-cardiac troponin-T (1:150; Abcam), chicken polyclonal anti-Brdu (1:150; Abcam) and rabbit polyclonal anti-phosphohistone-3 (1:150; Abcam). In ventricular cells and cardiac tissue, the nucleus was identified with 4′,6′-diamidino-2-phenylindole (DAPI, 1.5 μM; emission wavelength, 460 nm; Sigma-Aldrich) staining. Secondary antibodies used include goat anti-mouse IgG conjugated to conjugated to Alexa-555 (1:600–800; Life Technologies), goat anti-rabbit IgG conjugated to Alexa-647 (1:600–800; Life Technologies, Carlsbad, CA) and a goat anti-chicken IgG conjugated to Alexa-488 (1:600–800; Life Technologies). Non-specific staining was determined following the addition of the conjugated secondary antibody alone.

The Zeiss LSM Image Browser was used to calculate the fibrin clot length and fibrin clot area of two additional serial sections of each paraffin-fixed neonatal rat heart. Furthermore, the tissue sections were used to calculate the density of cycling neonatal rat ventricular cardiomyocytes. With regard to the viable region of the apex region, a maximum distance of ∼500 µm from the fibrin clot (not including the region immediately adjacent to the fibrin clot) was examined to quantitate the number of cycling ventricular cardiomyocytes and the data was normalized to an area of 0,2 mm^2^. The entire length of the region immediately adjacent to the fibrin clot was examined to quantitate the number of nestin^(+)^-ventricular cardiomyocytes that re-entered the cell cycle and the data normalized to an area of 0,2 mm^2^.

### Western blot

Protein lysates of ventricular cells was prepared and subjected to SDS-electrophoresis, as previously described^15,16,20^. Antibodies used include the mouse monoclonal anti-nestin (1:500; Chemicom), mouse monoclonal anti-p38α MAPK (1:1000; Santa Cruz Biotechnology, Santa Cruz, CA), rabbit polyclonal directed against the threonine^180^/tyrosine^182^ residues of anti-p38 MAPK (1:1000; Cell Signalling Technology, Danvers, MA) rabbit polyclonal anti-heat shock protein 27 (HSP27; 1:500; Enzo Life Sciences, Farmingdale, NY), rabbit polyclonal directed against the serine^82^ residue of HSP27 (1:500; Cell Signalling Technology) and a mouse monoclonal anti-GAPDH (1:50,000; Ambion, Austin TX). Following overnight incubation at 4 °C, the appropriate secondary antibody-conjugated to horseradish peroxidase (1:20,000, Jackson Immunoresearch, West Grove, PA) was added and bands visualized utilizing the ECL detection kit (Perkin Elmer, Waltham, MA). Films were scanned with Image J software® and the target protein signal was depicted as arbitrary light units.

### Statistics

Data was presented as the mean ± S.E.M, (n) represents the number of independent preparations of neonatal rat ventricular cells and/or neonatal rat pups used per experiment. Data was evaluated by a student’s unpaired t-test or a one-way ANOVA (Origin 2016 32 bit; Northhampton, MA) and a significant difference determined by the Bonferroni post-hoc test and a value of P < 0.05 considered statistically significant.

## Supplementary information

Supplemental Legend and data

## Data Availability

Materials, data and associated protocols of the present paper are available to readers.
