# Supplementary material for: p38α MAPK inhibition translates to cell cycle re-entry of neonatal rat ventricular cardiomyocytes and de novo nestin expression in response to thrombin and after apex resection
Source: Sci Rep. 2019 Jun 3;9:8203. doi: 10.1038/s41598-019-44712-3 (PMC6547723; doi:10.1038/s41598-019-44712-3)

p38α MAPK inhibition translates to cell cycle re-entry of neonatal rat ventricular cardiomyocytes and de novo nestin expression in response to thrombin and after apex resection

VANESSA HERTIG[1], ANDRA BREZAI[1], ALEXANDRE BERGERON[1], LOUIS VILLENEUVE[1], MARC-ANTOINE GILLIS[1], AND ANGELINO CALDERONE[1,2*]

[1]*Montreal Heart Institute, Université de Montréal, Montréal, Québec, Canada;*

[2]*Département de Pharmacologie et Physiologie, Université de Montréal, Montréal, Québec, Canada*

[*]*Corresponding author*

Supplemental Figure 1. **Cell cycle re-entry and *de novo* nestin expression in neonatal rat ventricular cardiomyocytes (NNVMs)**. (**A**) Three day treatment of cardiac troponin-T$^{(+)}$-NNVMs (green fluorescence) with phorbol 12,13-dibutyrate (PDBu; 100 nM) failed to increase cell cycle re-entry as depicted by bromodeoxyuridine incorporation (BrdU; grey fluorescence) or induce nestin (red fluorescence) expression. (**B**) The co-treatment with PDBu and the p38α/β MAPK inhibitor SB203580 (10 μM) promoted cycle re-entry of NNVMs depicted by BrdU incorporation and *de novo* nestin expression. See ***Figure 2*** for quantitative analysis of the data.

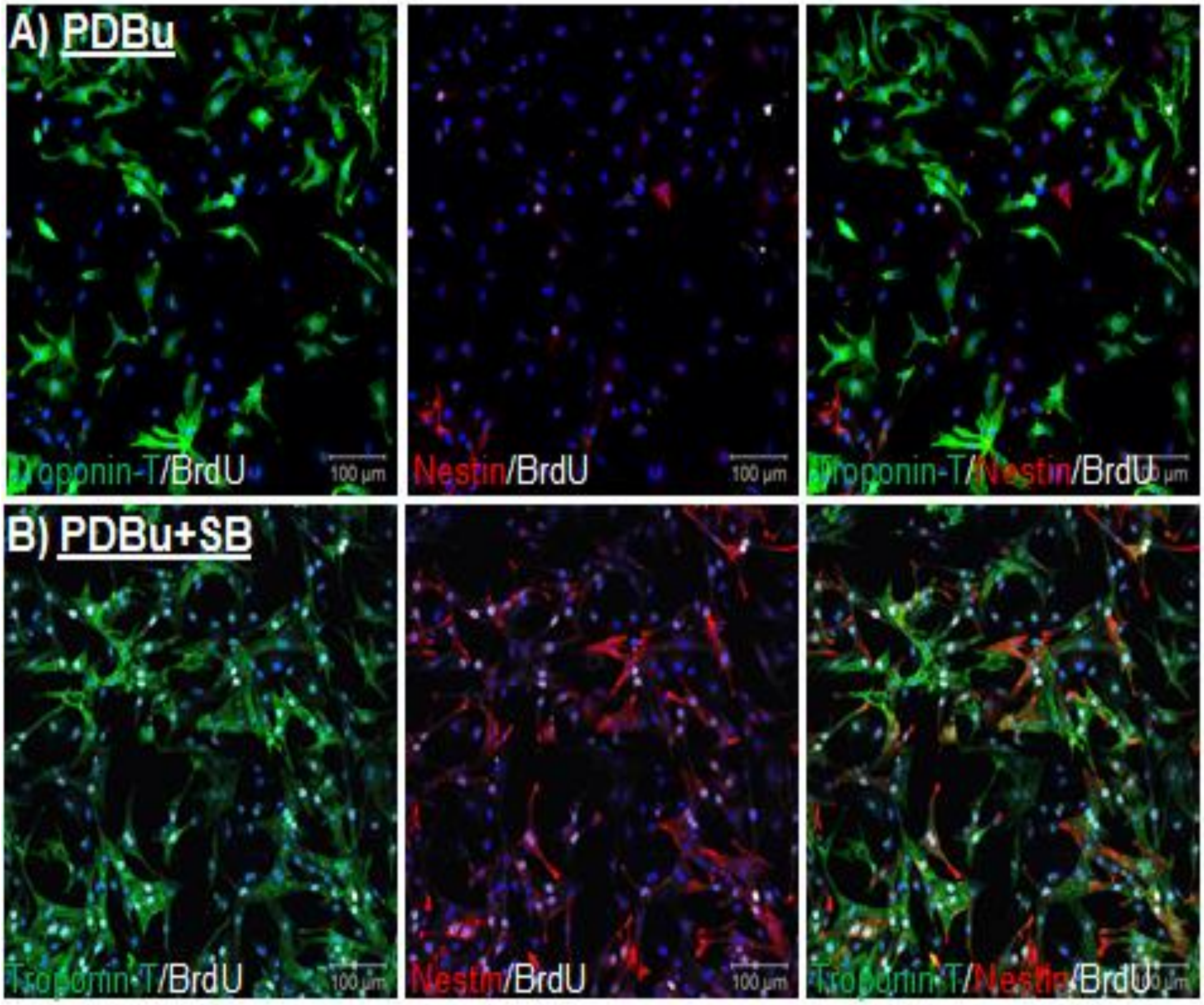

<u>Supplemental Figure 2.</u> Collagen type 1 staining of neonatal rat ventricular fibroblasts. In untreated neonatal ventricular cells, nestin staining (red fluorescence) was identified in collagen type $I^{(+)}$-neonatal rat ventricular fibroblasts (green fluorescence).

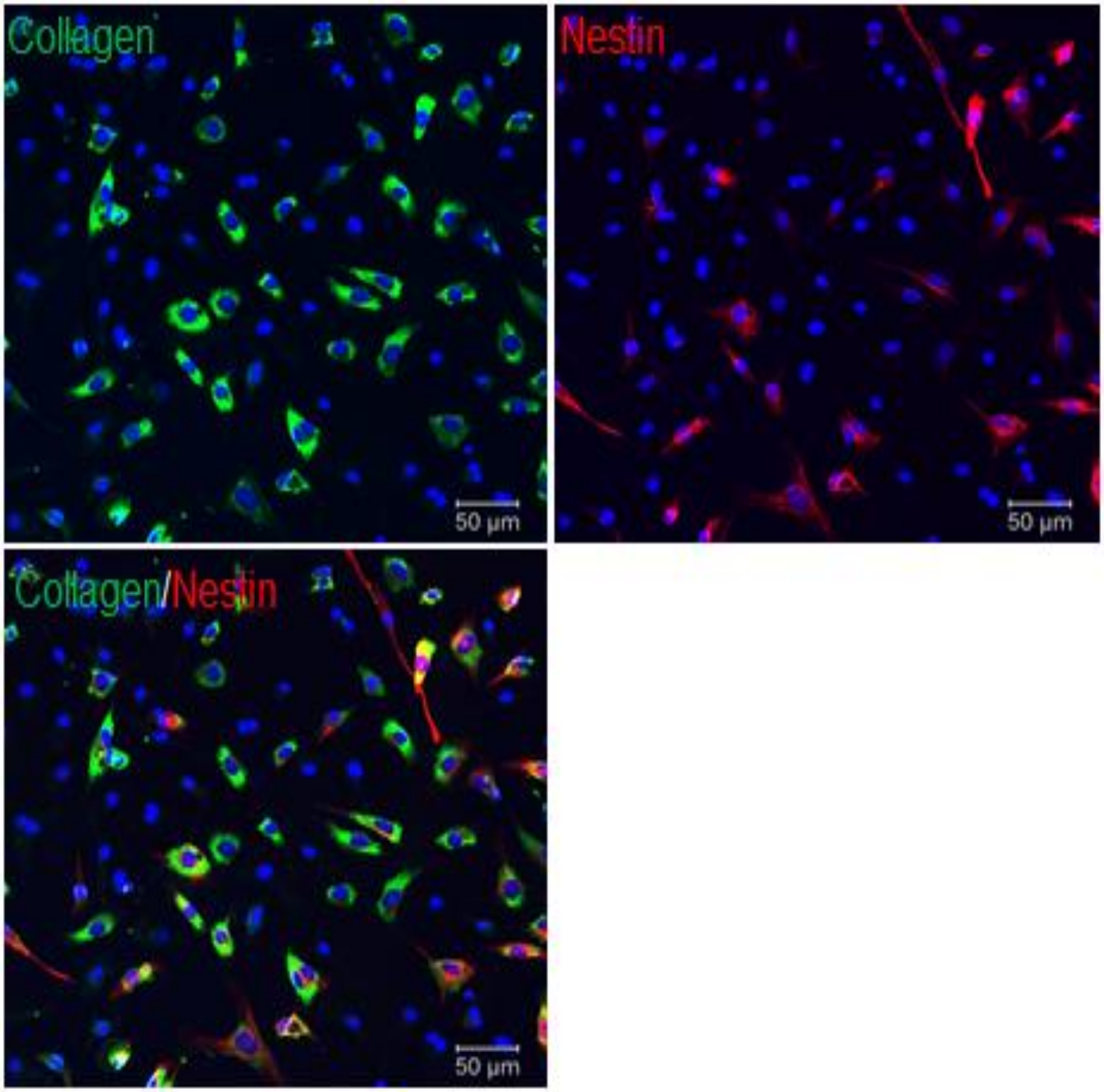

Supplement: Supplementary file 1 — Supplemental Legend and data [file 41598_2019_44712_MOESM1_ESM.pdf]
